# An Inactive Geminin Mutant That Binds Cdt1

**DOI:** 10.3390/genes6020252

**Published:** 2015-05-15

**Authors:** Marissa Suchyta, Benoit Miotto, Thomas J. McGarry

**Affiliations:** 1 Department of Medicine, Feinberg Cardiovascular Research Institute, Feinberg School of Medicine, Northwestern University Chicago, IL 60610, USA; E-Mail: msuchyta@gmail.com; 2 Epigenetics and Cell Fate, Sorbonne Paris Cité, University Paris Diderot, UMR 7216 CNRS, Paris 75013, France; E-Mail: benoit.miotto@paris7.jussieu.fr; 3 George Wahlen Veterans Affairs Medical Center, Room 2E 24, 500 Foothill Drive, Salt Lake City, UT 84103, USA

**Keywords:** DNA replication, pre-replication complex, Cdt1, geminin, HBO1

## Abstract

The initiation of DNA replication is tightly regulated in order to ensure that the genome duplicates only once per cell cycle. In vertebrate cells, the unstable regulatory protein Geminin prevents a second round of DNA replication by inhibiting the essential replication factor Cdt1. Cdt1 recruits mini-chromosome maintenance complex (MCM2-7), the replication helicase, into the pre-replication complex (pre-RC) at origins of DNA replication. The mechanism by which Geminin inhibits MCM2-7 loading by Cdt1 is incompletely understood. The conventional model is that Geminin sterically hinders a direct physical interaction between Cdt1 and MCM2-7. Here, we describe an inactive missense mutant of Geminin, Geminin^AWA^, which binds to Cdt1 with normal affinity yet is completely inactive as a replication inhibitor even when added in vast excess. In fact, Geminin^AWA^ can compete with Geminin^WT^ for binding to Cdt1 and prevent it from inhibiting DNA replication. Geminin^AWA^ does not inhibit the loading of MCM2-7 onto DNA *in vivo*, and in the presence of Geminin^AWA^, nuclear DNA is massively over-replicated within a single S phase. We conclude that Geminin does not inhibit MCM loading by simple steric interference with a Cdt1-MCM2-7 interaction but instead works by a non-steric mechanism, possibly by inhibiting the histone acetyltransferase HBO1.

## 1. Introduction

DNA replication is rigorously controlled such that the genome duplicates exactly once and only once per cell cycle [1]. To accomplish this task, the replication process is separated into two distinct and sequential steps. In the first step, called “licensing”, replication proteins assemble on the DNA at origins of replication to form a pre-replication complex or pre-RC. These proteins include the origin recognition complex (ORC), which binds directly to the DNA, the mini-chromosome maintenance complex (MCM2-7), which is part of the replication helicase, and the regulatory factors Cdc6 and Cdt1. In the second step, initiation, pre-RC disassembles, and the polymerization of nucleotides begins. The two steps occur at two different points in the cell cycle: licensing takes place during G1 phase, and initiation takes place during S phase. Importantly, licensing and initiation are mutually exclusive: initiation does not occur while the licensing process is ongoing, and licensing does not take place once replication has initiated. This separation of licensing and initiation into two distinct and non-overlapping phases assures that the genome duplicates itself only once. Passage of the cell through mitosis re-sets the system and allows pre-RC to assemble again in the next G1 phase.

Different types of cells use multiple different mechanisms to insulate licensing from initiation. In metazoan cells, the dominant mechanism involves the regulatory proteins Cdt1 and Geminin. Cdt1 is an essential component of pre-RC that is required to incorporate the MCM2-7 helicase into the complex [2]. After initiation, chromatin-bound Cdt1 is rapidly destroyed by ubiquitin-dependent proteolysis in a reaction that requires the ubiquitin-protein ligase Cul4-Ddb1^Cdt2^ and the chromatin-bound replication processivity factor PCNA [3,4]. This degradation step comprises one mechanism that blocks re-licensing after initiation. The second mechanism involves the unstable regulatory protein Geminin [5]. Geminin binds to Cdt1 and inhibits the incorporation of MCM complex into pre-RC [6,7]. Overexpression of Geminin completely inhibits DNA replication, both *in vitro* and *in vivo*. The concentration of Geminin fluctuates throughout the cell cycle [5]. It is absent during G1 phase, when licensing occurs, and it accumulates during S, G2 and M phases, when licensing is prohibited. Geminin is itself destroyed by ubiquitin-dependent proteolysis at the metaphase/anaphase transition in a reaction that requires the ubiquitin-protein ligase APC^Cdc20^. Cdt1 proteolysis and Geminin accumulation work in parallel to prevent re-licensing after initiation. Abolition of either process by itself does not measurably affect the extent of DNA replication, but if both processes are inactivated simultaneously then the genome is massively over-duplicated [4,8,9].

The molecular mechanism by which Geminin inhibits Cdt1 and prevents the incorporation of MCM complex into pre-RC is incompletely understood. The physical interaction between Geminin and Cdt1 is necessary in order for Geminin to inhibit MCM2-7 loading [6,7,10]. On the basis of the crystal structure of the Geminin-Cdt1 complex and *in vitro* binding studies using purified proteins, it was proposed that the amino terminal portion of Geminin binds to Cdt1 and that the carboxy terminal tail of Geminin sterically hinders a direct physical interaction between Cdt1 and the MCM complex [11,12]. More recent data suggest that the mechanism of Geminin’s action is more complex. In some experimental systems Geminin does not inhibit the incorporation of MCM complex into pre-RC, but rather prevents the formation of a more stable form of pre-RC in a reaction that requires ATP [13]. There is also evidence that Geminin affects the activity of replication origins by inhibiting the activity of histone acetylase HBO1, a co-activator of Cdt1 which is required for licensing [14,15]. Recruitment of HBO1 by Cdt1 in G1 phase may favor the opening of the chromatin structure around origins [15,16] or regulate the post-translational level of replication initiation factors [17].

Here we describe a missense mutant of Geminin that binds Cdt1 normally yet inhibits neither the incorporation of MCM complex into pre-RC nor DNA replication. In the presence of this mutant, the genome is vastly over-replicated. Our results argue against a model where Geminin inhibits MCM loading by sterically inhibiting a direct Cdt1-MCM interaction.

## 2. Materials and Methods

### 2.1. Plasmids

The construction of plasmids expressing Xenopus Geminin^WT^, Geminin^AWA^, Geminin^RTGG^ and Geminin^KKFEV^ was previously described [10]. To construct a plasmid expressing mouse Geminin^WT^, the full-length mouse Geminin coding sequence from IMAGE clone 833335 was amplified by PCR and inserted between the BamHI and EcoRI sites of pET Duet 1 (Novagen). The YWK sequence in this plasmid was mutated to AWA by QuikChange site-directed mutagenesis (Stratagene). To construct plasmids expressing Cdt1:Geminin complexes, full-length Cdt1 and Geminin coding sequences were amplified by PCR and inserted together into pET Duet 1, either between either the NdeI and KpnI sites (Cdt1) or the BamHI and EcoRI sites (Geminin). In this construct, the Geminin gene was fused to an amino-terminal hexa-histidine tag, while the Cdt1 gene was untagged. The parent Xenopus Cdt1 gene was the kind gift of Dominic Maiorano [2], and the parent mouse Cdt1 gene was a pCMV-Sport6.1.ccdb clone obtained from Invitrogen. For isothermal calorimetry, genes encoding mouse Cdt1^172–368^ and mouse Geminin^79–157^ were amplified by PCR and inserted separately into pET Duet1 between either the NdeI and KpnI sites (Cdt1) or the BamHI and EcoRI sites (Geminin). For MCM binding studies, the full-length mouse Cdt1 coding sequence was inserted between the XhoI and XbaI sites of pCS2-MT. All plasmids were sequenced to ensure that secondary mutations had not been introduced.

### 2.2. Protein Expression

Plasmids encoding hexa-histidine-tagged proteins were expressed in E. coli strain BL21 and purified over nickel-NTA agarose using standard techniques (Qiagen). Protein concentrations were measured using the Bradford Assay (Bio-Rad) and confirmed by gel electrophoresis and coomassie blue staining. For isothermal calorimetry, the proteins were dialyzed against 20 mM Na-HEPES pH 7.5, 300 mM NaCl and 7 mM β-mercaptoethanol. Protein concentrations were determined by direct ultraviolet absorbance using a spectrophotometer (Thermo Scientific NanoDrop 2000c).

### 2.3. Replication Reactions

DNA replication extracts from Xenopus eggs were prepared as previously described [18], except that cycloheximide (100 µg/mL) was added to all buffers that the eggs came in contact with after the dejellying step and the eggs were incubated at room temperature for 15 minutes after ionophore activation to ensure maximal degradation of endogenous Geminin. Cycloheximide (200 µg/mL) was added to all replication extracts before use. Replication reactions, density substitution experiments and immunodepletion of Cdt1 and Geminin from egg extracts were carried out as previously described [8,18]. All animal experiments were done in accordance with a protocol approved by the Northwestern University Animal Care and Use Committee.

### 2.4. In Vitro Binding Studies

The binding of recombinant Geminin to *in vitro*-translated Myc-Cdt1 was measured as described [10]. To measure the effect of Geminin on MCM binding to Cdt1, reactions contained 16 µL of reticulocyte lysate containing *in vitro*-translated mouse Myc-Cdt1, 1 µL of purified mouse MCM4/6/7 complex (50 ng, the kind gift of Hisao Masai [19]) and 5 µL of recombinant Geminin diluted in 10 mM HEPES pH 7.6/300 mM NaCl. Reactions containing Geminin and Myc-Cdt1 were incubated on ice for 45 minutes before adding MCM 4/6/7 complex. After a further incubation for 45 minutes, anti-Myc 9E10 antibodies conjugated to sepharose (Santa Cruz Biotechnology SC-40 AC) were added and the mixture was tumbled at 4 °C for 1 hour. Antibody-protein complexes were washed first with PBS then with IP buffer (50 mM β-glycerol phosphate pH 7.4, 5 mM EDTA, 0.1% triton X-100 and 150 mM NaCl) before gel electrophoresis. The interaction between Geminin, HA-tagged Cdt1, and FLAG-tagged HBO1 was determined as described previously [14].

### 2.5. In Vivo Chromatin Binding Studies

Replication extracts were depleted of endogenous Geminin then supplemented with recombinant Geminin^WT^ or Geminin^AWA^ and template sperm DNA. Individual reactions (10 µL) were incubated at room temperature until nuclear assembly was complete as judged by Hoechst 33342 staining (about 30 minutes, [18]). The reactions were then diluted with 60 µL ice-cold egg lysis buffer (ELB; 10 mM K-HEPES pH 7.7, 50 mM KCl, 2.5 mM MgCl_2_, 250 mM sucrose, 1 mM dithiothreitol) and placed on ice. To isolate chromatin, the mixture was layered atop a cushion of 170 µL ELB containing 500 mM sucrose and spun at 10,000× *g* for 15 minutes. The supernatant was aspirated and the pellet was resuspended in protein electrophoresis sample buffer (50 mM Tris-HCl pH 6.8, 2% SDS, 10% glycerol, 1% β-mercaptoethanol, 12.5 mM EDTA and 0.02% bromophenol blue).

### 2.6. Isothermal Calorimetry

Isothermal calorimetry was performed by the Keck Biophysics Facility at Northwestern University using a MicroCal ITC200 isothermal titration calorimeter (GE Healthcare) as described [12]. Briefly, purified proteins were extensively dialyzed against 20 mM Na-HEPES pH 7.5, 300 mM NaCl and 7 mM β-mercaptoethanol. Experiments were performed at 18 °C, and each experiment consisted of 17 injections of 2.4 µL of Geminin^WT^ or 2.5 µL of Geminin^AWA^ into 200 µL Cdt1 solution. The concentrations used were Geminin^WT^ 0.432 mM, Geminin^AWA^ 0.297 mM and Cdt1 0.044 mM. As a control, proteins were injected into buffer; the heat of dilution was found to be negligible.

### 2.7. HBO1 Acetylation Assays

The acetylation of histone H4 by HBO1 was measured as described previously [14,15,28].

### 2.8. Antibodies

Polyclonal antibodies raised against Xenopus Geminin and Xenopus Cdt1 were described previously [5,8]. Antibodies to mouse MCM4 were kindly supplied by Hisao Masai, and antibodies to Xenopus MCM3 were kindly supplied by Ron Laskey. Anti-Myc 9E10 antibodies (Santa Cruz sc-40), anti-HA F-7 antibodies (Santa Cruz sc-7392), anti-FLAG M2 antibodies (Sigma-Aldrich F1804) and anti-histone H4 K12Ac antibodies (Upstate Biotechnology) were purchased from the manufacturer.

## 3. Results

### 3.1. Geminin^AWA^ Binds Cdt1, Yet Fails to Inhibit DNA Replication

**Figure 1:**
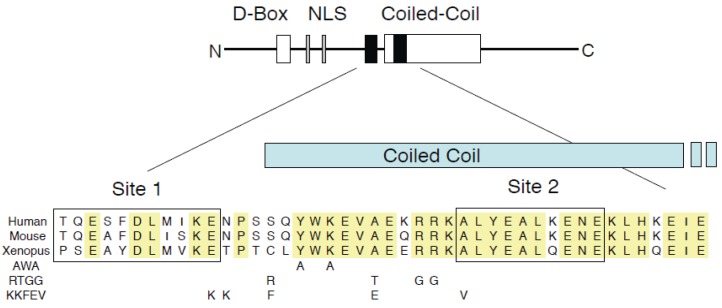
Structure and sequence of Geminin. (Top) Diagrammatic representation of a Geminin monomer. Black bars, Cdt1 binding sites; D-box, destruction box required in cis for ubiquitylation; NLS, nuclear localization signal. (Bottom) Sequence alignment of Human, mouse and Xenopus Geminin. Conserved residues are shaded. Mutated residues in Geminin^AWA^, Geminin^RTGG^ and Geminin^KKFEV^ are indicated.

The active form of Geminin is a dimer in which the two Geminin monomers are held together in parallel by a coiled-coil domain consisting of eight heptad repeats (Figure 1, [10,20]). Only the coiled-coil and the sequences immediately amino-terminal to it are required to inhibit licensing [5]. Geminin has two binding sites for Cdt1 that have been defined both by X-ray crystallography and by mutagenesis studies [10,12,20,21]. Site 1 encompasses amino acids 79–89 of Xenopus Geminin and is located immediately amino-terminally to the coiled coil (Figure 1). Site 2 encompasses amino acids 117–129 and is located within the coiled coil. Site 2 is quantitatively more important for Cdt1 binding; mutation of Site 2 is sufficient to eliminate any measureable Geminin-Cdt1 interaction *in vitro*, and the mutant protein does not inhibit DNA replication even when present in excess [12]. In contrast, deleting Site 1 reduces the Geminin-Cdt1 association constant by about 10-fold, and the mutant protein still retains replication-inhibition activity when present at a high concentration [12]. Geminin mutants that do not dimerize or do not bind Cdt1 are inactive [10].

**Figure 2:**
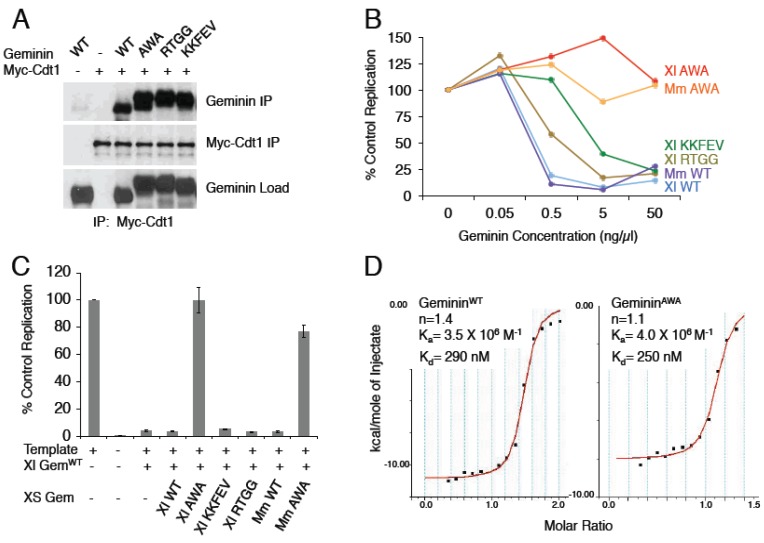
Defective Geminin mutants bind Cdt1. (**A**) Geminin missense mutants bind Cdt1. Recombinant Xenopus Geminin proteins were mixed with translated Myc-Cdt1. The reactions were precipitated with anti-Myc antibody, and the precipitate was blotted with the indicated antibodies. (**B**) Inhibition of DNA replication by Geminin mutants. Recombinant Geminin proteins were added to replication extracts made from Xenopus eggs at the indicated concentrations, and the extent of sperm DNA replication was measured. Average of two experiments. (**C**) Geminin^AWA^ competes away Geminin^WT^. Recombinant Geminin^WT^ (2 ng/µL) and an excess of a Geminin mutant (50 ng/µL) were added to replication extracts, and the extent of DNA replication was measured. Average of two experiments. (**D**) Geminin^AWA^ and Geminin^WT^ have a similar binding affinity for Cdt1. The binding affinity between recombinant mouse Cdt1^172–368^ and either WT or AWA mouse Geminin^72–157^ was measured by isothermal calorimetry.

In a previous study, we heavily mutagenized Geminin’s Cdt1 binding region and isolated three missense mutants that map to the area between the two Cdt1 binding sites (Figure 1): Geminin^AWA^, Geminin^KKFEV^ and Geminin^RTGG^ [10]. As expected, all three mutant proteins bind to Cdt1 in co-precipitation assays (Figure 2A and [10]). These three mutants, however, do not inhibit DNA replication to the same extent as wild-type Geminin (Figure 2B). This is surprising, since a steric hindrance model of Geminin action would predict that any Geminin mutant capable of binding to Cdt1 would inhibit MCM loading as long as its C-terminal tail was intact.

One possible explanation of the results is that the mutants bind Cdt1 with a reduced affinity. To test this possibility, we added increasing concentrations of different recombinant Geminin proteins to DNA replication extracts made from Xenopus eggs (Figure 2B). Egg extracts faithfully reproduce the replication process that occurs *in vivo*. When permeabilized sperm heads are added as a template, the chromatin de-condenses and becomes surrounded by a normal nuclear membrane [22]. The DNA is then replicated completely and exactly once [23]. In the experiment shown, wild-type Geminin completely extinguishes DNA replication when added to a concentration of 0.5 ng/µL (10 nM). Two of the mutants, Geminin^KKFEV^ and Geminin^RTGG^, do not completely inhibit DNA replication at this concentration but do so at a concentration ~10-fold higher, suggesting that they have a reduced binding affinity for Cdt1. Geminin^AWA^, however, does not to inhibit DNA replication at any concentration, even when added to a concentration ~100-fold higher (1 µM). Both Xenopus and mouse Geminin^AWA^ exhibit this property. These results suggest that Geminin^AWA^ either binds to Cdt1 extremely weakly or does not inhibit DNA replication even when it is bound to Cdt1.

To distinguish between these possibilities, we performed a competition assay (Figure 2C). Geminin^WT^ was added to Xenopus egg extract at a concentration sufficient to inhibit DNA replication completely, then a 25-fold excess of Geminin^RTGG^, Geminin^KKFEV^ or Geminin^AWA^ was added before starting the replication reaction. If a given mutant binds weakly to Cdt1, then it would either not compete effectively with wild-type Geminin or, if it did compete, it would inhibit Cdt1 itself. In contrast, if a given mutant does not inhibit replication when bound to Cdt1, it could compete with wild-type Geminin for access to Cdt1 and restore replication to normal. We found that the addition of excess Geminin^AWA^ restored DNA replication to normal levels, while the addition of excess Geminin^RTGG^ or Geminin^KKFEV^ had no effect. These results support the contention that Geminin^RTGG^ and Geminin^KKFEV^ have a reduced binding affinity for Cdt1 while Geminin^AWA^ is defective in some other way.

Finally, we directly compared the binding affinity of mouse Geminin^WT^ and Geminin^AWA^ for Cdt1 by isothermal calorimetry. We found that the two proteins had about the same dissociation constant (290 vs 250 nM), indicating that they bind Cdt1 with near equal affinity (Figure 2D). We conclude that Geminin^AWA^ does not inhibit DNA replication even when bound to Cdt1.

### 3.2. Geminin^AWA^ Allows MCM Loading In Vivo

**Figure 3:**
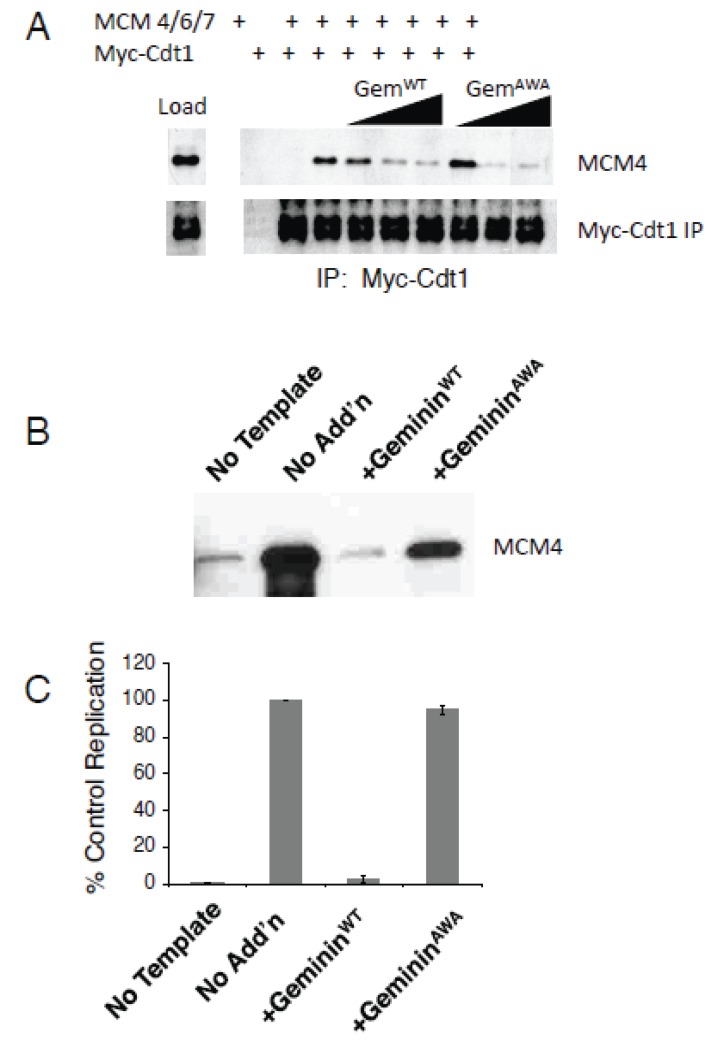
Geminin^AWA^ inhibits MCM loading *in vitro* but not *in vivo*. (**A**) Geminin^AWA^ inhibits the MCM ←→Cdt1 interaction *in vitro*. Increasing concentrations of recombinant Geminin protein (5 μg/mL, 10 μg/mL, or 40 μg/mL) were mixed with translated Myc-Cdt1. After 40 min, purified MCM4/6/7 complex was added. After another 45 min, the reaction was incubated with anti-Myc antibody, and the precipitate was blotted for the proteins shown. (**B**, **C**) Geminin^AWA^ does not inhibit MCM loading *in vivo*. (**B**) Replication extracts were depleted of endogenous Geminin with anti-Geminin antiserum, then either recombinant Geminin^WT^ or Geminin^AWA^ was added back (50 μg/mL). Permeabilized sperm heads were added, and nuclei were allowed to assemble. The chromatin was then pelleted through a sucrose cushion, and the amount of MCM4 in the precipitate was determined by immunoblotting. (**C**) In a separate aliquot of the reaction, DNA replication was allowed to proceed to completion and the extent of replication was measured. Average of two experiments

Our results argue against a model where Geminin inhibits MCM loading by direct steric interference. It has been previously shown, however, that purified Cdt1 and MCM proteins directly associate with one another *in vitro* and that Geminin inhibits this interaction [11,12]. To see if Geminin^AWA^ inhibits the Cdt1-MCM association *in vitro*, we added different concentrations of recombinant Geminin^WT^ or Geminin^AWA^ to a mixture of purified MCM4/6/7 complex and *in vitro*-translated Myc-Cdt1. The Cdt1 was immunoprecipitated using anti-Myc antibody, and the amount of MCM4 in the precipitate was determined by immunoblotting. We found that Geminin^WT^ and Geminin^AWA^ both inhibited the interaction between Cdt1 and MCM4/6/7 *in vitro* at about the same concentration (Figure 3A). This result is at odds with our observation that Geminin^AWA^ does not inhibit DNA replication *in vivo*.

We next tested whether Geminin^AWA^ inhibits the loading of MCM complex onto chromatin in replication extracts. Recombinant Geminin^WT^ or Geminin^AWA^ was added to replication extracts at a high concentration (50 ng/µL), and the replication reaction was initiated by adding demembranated sperm. Pre-replication complexes were allowed to assemble on chromatin for 30 minutes, then the replication process was arrested by diluting the reaction mixture six-fold with ice-cold buffer and placing it on ice. The chromatin was isolated by pelleting it through a sucrose cushion, and the amount of chromatin-associated MCM4 protein in the pellet was determined by immunoblotting. Adding Geminin^WT^ reduced the amount of chromatin-bound MCM4 down to background levels, while adding Geminin^AWA^ still allowed a substantial amount of MCM loading (Figure 3B). In a separate aliquot of the same reaction mixture, DNA replication was allowed to proceed to completion. In the presence of Geminin^AWA^, the total amount [^32^P]-dATP incorporated into DNA was normal, while Geminin^WT^ inhibited [^32^P]-dATP incorporation completely (Figure 3C). We conclude that Geminin^AWA^ does not inhibit the incorporation of MCM complex into pre-RC *in vivo,* and that the *in vitro* binding studies using purified proteins do not entirely reflect their biological interactions.

### 3.3. Geminin^AWA^ Does Not Inhibit a Second Round of Replication

**Figure 4:**
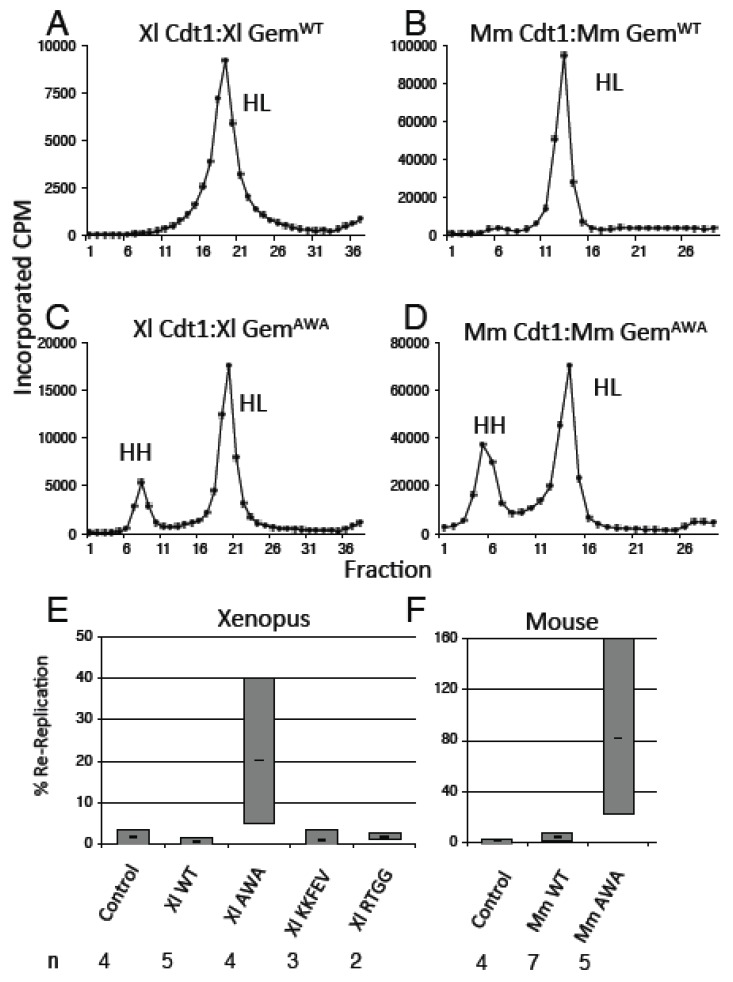
Geminin^AWA^ does not inhibit re-replication. Replication extracts made from Xenopus eggs were depleted of both Geminin and Cdt1 using specific antibodies, then supplemented with recombinant Geminin:Cdt1 complexes. Replication was allowed to progress in the presence of BrdUTP, then the density of the product was determined by equilibrium ultracentrifugation. (**A**–**D**) Typical gradient profiles; (**E**) extent of re-replication for different Geminin mutants. The gray bar indicates the range, and the horizontal line indicates the mean. n, number of experiments.

Having established that Geminin^AWA^ does not inhibit DNA replication when added in excess, we next performed density label experiments to determine whether the mutant would allow a second round of DNA replication within a single S phase. To test the effects of Geminin mutants, we depleted both Geminin and Cdt1 from replication extracts using specific antibodies then added back recombinant Cdt1:Geminin complexes purified from bacteria [24]. As previously discussed, disabling the Geminin-dependent mechanism by itself will not permit a second round of replication; Cdt1 degradation must also be circumvented [4,5]. To obviate the effect of Cdt1 degradation, the complex was added to an ~8-fold molar excess (~200 nM) compared to the endogenous Cdt1 concentration (~25 nm, [4]). The extract was supplemented with α-[^32^P]-dATP, the density label BrdUTP, and cycloheximide to ensure that cyclin synthesis did not drive the extract into mitosis [25]. Demembranated sperm were added as the template, and DNA replication was allowed to proceed to completion. The radioactive product DNA was purified and analyzed by equilibrium gradient centrifugation to measure the amount of doubly-replicated (heavy-heavy) DNA. In untreated extracts or extracts supplemented with Cdt1:Geminin^WT^, Cdt1:Geminin^KKFEV^ or Cdt1:Geminin^RTGG^, the product of replication consisted entirely of heavy-light DNA with virtually no heavy-heavy DNA (Figure 4A,B,E), indicating that a single round of semiconservative replication had occurred. In contrast, in extracts supplemented with Cdt1:Geminin^AWA^, the product contained a substantial amount of heavy-heavy DNA (Figure 4C–E), indicating that more than one round of DNA synthesis had taken place. The amount of heavy-heavy DNA was greater in extracts supplemented with mouse Cdt1:Geminin^AWA^ than in extracts supplemented with Xenopus Cdt1:Geminin^AWA^ (25%–150% *vs*. 25%–40%; Figure 4E). These results indicate that Geminin^AWA^ is defective in its ability to suppress a second round of DNA replication during the S phase.

### 3.4. Geminin^AWA^ Does Not Inhibit the Histone Acetyltransferase HBO1

**Figure 5:**
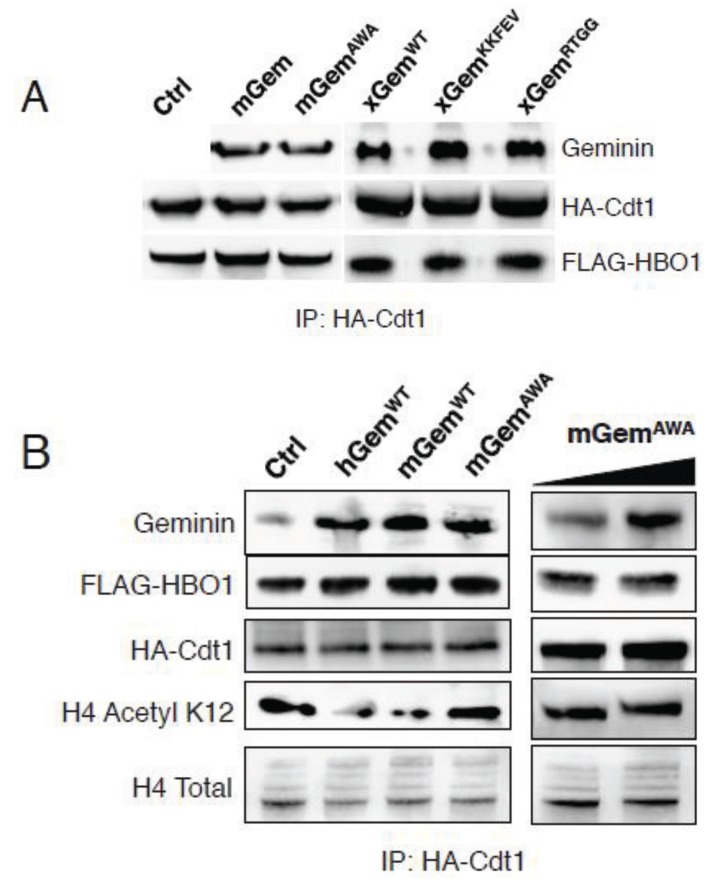
Geminin^AWA^ does not inhibit HBO1 acetyltransferase activity. (**A**) Geminin^AWA^ forms a ternary complex with Cdt1 and HBO1. HA-Cdt1:FLAG-HBO1 complex was precipitated from HeLa cells expressing both proteins using anti-HA antibody. The precipitate was incubated with recombinant Geminin, and after extensive washing the amount of Geminin incorporated into the complex was determined by immunoblotting. (**B**) Geminin^AWA^ does not inhibit HBO1. HA-Cdt1:FLAG-HBO1 complex was purified using anti-HA antibody as in part (A), eluted with HA peptide, then incubated with acetyl coenzyme A, histone H4 peptide, and different recombinant Geminin proteins. The amount of acetylated histone H4 peptide was determined by immunoblotting.

Because Geminin^AWA^ is inactive as a replication inhibitor yet has intact steric properties, we examined other activities of Geminin that might be deficient in the mutant. HBO1 (histone acetyltransferase bound to ORC1) is a histone acetyltransferase that interacts with ORC1, MCM2 and Cdt1 and serves as a co-activator of Cdt1 [14,26,27]. Geminin forms a ternary complex with HBO1 and Cdt1 *in vitro* and in that context inhibits HBO1’s acetyltransferase activity [15]. To test whether Geminin^AWA^ could form the ternary complex, protein lysates of HeLa cells expressing HA-Cdt1 and FLAG-HBO1 were precipitated with anti-HA antibody and the precipitate was incubated with recombinant mouse Geminin^WT^ or Geminin^AWA^. After extensive washing, the amount of Geminin bound to the beads was determined by immunoblotting. Both Geminin^WT^ and Geminin^AWA^ formed a ternary complex with Cdt1 and HBO1 (Figure 5A). To see if Geminin^AWA^ inhibited HBO1 acetyltransferase activity, immunoprecipitated HA-Cdt1:Flag-HBO1 complexes were eluted off anti-HA beads and incubated with recombinant Geminin proteins in the presence of acetyl coenzyme A and a histone H4 peptide [14,28]. The amount of K12-acetyl histone H4 produced was determined by immunoblotting (Figure 5B, left panel). Both human and mouse wild-type Geminin strongly inhibited the acetyltransferase activity of HBO1 while mouse Geminin^AWA^ did not, even when added to three-fold the concentration of wild-type Geminin (Figure 5B, right panel). These results suggest that the primary defect in Geminin^AWA^ is an inability to inhibit the acetyltransferase activity of HBO1.

## 4. Discussion

In this paper we challenge the conventional model that Geminin inhibits replication licensing by sterically hindering a physical interaction between the licensing factor Cdt1 and the replication helicase MCM2-7. We describe an inactive mutant of Geminin, Geminin^AWA^, that binds to Cdt1 with normal affinity yet does not inhibit the incorporation of MCM complex into pre-RC. Geminin^AWA^ does not inhibit DNA replication when present in excess, nor does it prevent a second round of DNA replication when present at a more physiological concentration. Geminin^AWA^ effectively competes with wild-type Geminin for access to Cdt1 and reverses the inhibitory effect of wild-type Geminin on DNA replication. The phenotype of Geminin^AWA^ is incompatible with a steric hindrance model. We furthermore show that Geminin^AWA^ does not inhibit the enzymatic activity of the histone acetyltransferase HBO1, raising the possibility that Geminin inhibits replication licensing by inhibiting this enzyme.

The YWK motif that is mutated in Geminin^AWA^ is highly conserved among vertebrates ranging from humans to medaka fish (Figure 1). In Drosophila the corresponding sequence is YYK, and in Anopheles it is YWE. We have found that Geminin^Y106F^ has the same activity as wild-type Geminin, which makes it unlikely that Geminin is regulated by Y106 phosphorylation [10]. The YWK sequence is incorporated into the first heptad repeat of Geminin’s coiled-coil, but probably does not disrupt the protein’s quaternary structure because the two mutated residues, Y106 and K108, are not involved in the coil-to-coil interaction and are exposed to the solvent [20]. It also seems unlikely that the AWA mutation rotates the coiled-coil or changes its orientation in three-dimensional space. Indeed, it has been shown that the coiled-coil from GCN4 can be substituted for Geminin’s coiled-coil, and the fusion protein is still active as a replication inhibitor [12].

The steric hindrance model of Geminin action was based on the crystal structure of the Geminin-Cdt1 complex and *in vitro* binding studies using purified proteins. Several groups have shown that Cdt1 and components of the MCM complex interact *in vitro* and that Geminin inhibits this association [11,12,29]. We have found that Geminin^AWA^ inhibits the Cdt1-MCM interaction *in vitro* but does not abolish MCM2-7 loading *in vivo*. Apparently the *in vitro* binding assays do not completely reflect Geminin’s biological effects. We cannot totally exclude the possibility that Geminin acts in part by steric hindrance, since the amount of MCM loaded onto chromatin in the presence of Geminin^AWA^ was quantitatively reduced to about 25% of the amount loaded in untreated extracts (Figure 3B). In these experiments, however, the amount of Geminin^AWA^ added to the extract was about 25× the physiological concentration, which would exaggerate any subtle effect of steric hindrance. Our results clearly demonstrate that steric hindrance alone cannot entirely explain Geminin’s mechanism of action.

Steric hindrance is thought to be mediated by the carboxy-terminal portion of Geminin’s coiled-coil domain, because shortening this region with deletion mutations simultaneously destroys both the protein’s ability to inhibit the Cdt1-MCM interaction and its ability to inhibit replication [12]. Moreover, replacing the deleted sequences with a portion of the GCN4 coiled-coil restores both activities, indicating that the inhibitory activity is not sequence-specific. The carboxy-terminal part of the coiled-coil is not disrupted in Geminin^AWA^, so if this model were correct, we would expect Geminin^AWA^ to be fully active as a replication inhibitor. The phenotypes of the C-terminal Geminin mutations might possibly be explained by their effects on dimerization. Our laboratory previously demonstrated that dimerization of Geminin is required for activity and that dimerization is mediated through the coiled-coil domain [10]. The deletion mutations in the carboxy-terminal part of the coiled-coil may be inactive because they do not dimerize, and replacing these sequences with the GCN4 coiled-coil may restore dimerization.

Although the steric properties of Geminin^AWA^ are intact, we did find that the mutant protein is defective in its ability to inhibit the histone acetyltransferase HBO1. HBO1 associates with several components of pre-RC, including Cdt1, and knockdown of the protein in cultured cells causes decreased binding of the MCM complex to chromatin during G1 phase [27]. HBO1 is thought to acetylate histone H4 tails and induce a conformation of chromatin that is conducive to replication licensing. HBO1 is a co-activator of Cdt1; overexpression of HBO1 increases the amount of over-replication that occurs when Cdt1 is overexpressed, but overexpression of HBO1 by itself has no effect [14]. It is not clear whether HBO1 is essential for DNA replication in Xenopus egg extracts. It has been reported that extracts depleted of HBO1 with specific antibodies are unable to replicate DNA [17], but in these experiments it was not demonstrated that adding back HBO1 restored DNA replication. Thus, it remains possible that the anti-HBO1 antibodies depleted some other essential replication factor, perhaps Cdt1 itself. Although our results are consistent with the model that Geminin inhibits pre-RC assembly primarily by inhibiting HBO1, we cannot exclude other possibilities.

Several groups have reconstituted replication licensing *in vitro* using purified components, and their results may place constraints on the mechanism of Geminin action. It has been reported that Geminin inhibits licensing of sperm chromatin in a reaction that consists only of purified nucleoplasmin, ORC complex, Cdc6, Cdt1 and MCM2-7 complex [30]. HBO1 was not present in the licensing reaction, indicating that Geminin may inhibit licensing by a non-HBO1-dependent mechanism. More recently, it has been reported that incorporation of MCM complex into pre-RC on a plasmid origin requires only ORC complex, Cdc6, Cdt1 and ATP [13]. In this system, Geminin does not inhibit the recruitment of MCM complex to pre-RC. Geminin, in fact, becomes incorporated into pre-RC and inhibits its conversion to a more stable salt-resistant form in a reaction that requires ATP hydrolysis. In parallel, *in vivo* studies have detected two different populations of MCM complex interacting with chromatin with differing stability [31,32]. These results raise the possibility that the amino-terminal portion of Geminin might inhibit an intrinsic enzymatic activity of pre-RC that leads to its internal reorganization and stabilization. Consistent with this idea, the recent crystal structure of ORC bound onto DNA suggests that ORC switches between auto-inhibited and active conformations that might regulate its activity at different stages of the cell cycle [33]. Further studies will determine whether Geminin regulates this transition or some other rearrangement of pre-RC.

## 5. Conclusions

Geminin does not inhibit MCM loading by simple steric interference with a Cdt1-MCM2-7 interaction but instead works by a non-steric mechanism, possibly by inhibiting the histone acetyltransferase HBO1.
